# Supplementary material for: Spin-orbit enabled all-electrical readout of chiral spin-textures
Source: Nat Commun. 2022 Mar 24;13:1576. doi: 10.1038/s41467-022-29237-0 (PMC8948229; doi:10.1038/s41467-022-29237-0)
Supplement: Supplementary file 1 — Supplementary Information [file 41467_2022_29237_MOESM1_ESM.pdf]

Supplementary Materials

Spin-orbit enabled all-electrical readout of chiral spin-textures

Imara Lima Fernandes, Stefan Blügel and Samir Lounis

Supplementary Note 1 - Topological charge and helicity.

The topological charge $\mathcal{Q}$ characterizes the topological nature of magnetic spin-textures by measuring the wrapping of the magnetization unit vector $\mathbf{n}(\mathbf{r})$ around the unit sphere [1]:

$$\mathcal{Q} = \frac{1}{4\pi} \int\int d^2\mathbf{r} \quad \mathbf{n}(\mathbf{r}) \cdot \left[\frac{\partial \mathbf{n}(\mathbf{r})}{\partial x} \times \frac{\partial \mathbf{n}(\mathbf{r})}{\partial y} \right] . \tag{1}$$

Besides the topological charge, the helicity γ and vorticity m are very instrumental to distinguish spin-textures sharing the same $\mathcal{Q}$. As reminder, here we give their definition following Ref. [1]. Utilizing spherical coordinates, the magnetization field of the magnetic texture can be expressed as:

$$\mathbf{n}(\mathbf{r}) = (\cos\Phi(\varphi)\sin\Theta(\mathbf{r}), \sin\Phi(\varphi)\sin\Theta(\mathbf{r}), \cos\Phi(\mathbf{r})) \ , \tag{2}$$

where the position vector $\mathbf{r} = r(\cos\varphi, \sin\varphi)$ is given in polar coordinates. Then, by substituting $\mathbf{n}(\mathbf{r})$ into Supplementary Equation 1, we obtain:

$$\mathcal{Q} = -\left[\frac{\cos\Theta(r)}{2}\right]_{r=0}^{r=\infty} \left[\frac{\Phi(\varphi)}{2\pi}\right]_{\varphi=0}^{\varphi=2\pi} . \tag{3}$$

In general, the magnetic moment at the skyrmion's core points down, i.e., $\Theta(0) = \pi$, while the moments at the periphery of the magnetic structure point up, i.e., $\Theta(r \to \infty) = 0$. Thus, $\left[{\cos\Theta(r)}/{2}\right]_{r=0}^{r=\infty} = 1$. Meanwhile, the vorticity is defined as $m = \left[{\Phi(\varphi)}/{2\pi}\right]_{\varphi=0}^{\varphi=2\pi}$. Considering the boundary conditions at $r \to \infty$ fixed, the vorticity determines the topological charge as $\mathcal{Q} = -m$. In addition, the helicity can be defined as phase γ in

$$\Phi(\varphi) = m\varphi + \gamma. \tag{4}$$

Supplementary Note 2 - Multiple-scattering expansion of the LDOS.

We invoke multiple-scattering theory to demonstrate that upon rotation of magnetic moments at the neighborhood of a given atomic moment i, the local density of states (LDOS) will experience modifications intimately related to the electron scattering among the canted spin moments. Starting without loss of generality with the hamiltonian H of a non-magnetic system without spin-orbit coupling (SOC), the Green function is simply defined as $G(E) = (E - H)^{-1}$, from which the LDOS at a given site i can be extracted:

$$\mathrm{LDOS}_i(E) \;=\; -\frac{1}{\pi}\mathrm{Im}\,\mathrm{Tr}\,G_{ii}(E)\,, \tag{5}$$

where the trace is taken over the orbital and spin angular momenta indices.

If the atoms carry magnetic moments, the atomic potentials are modified by an additional $v_i = U_i(\mathbf{S}_i \cdot \boldsymbol{\sigma})$ for each site i, where U defines the atomic exchange splitting, while SOC gives rise to $v_i^{\mathrm{so}} = \lambda_i(\mathbf{L}_i \cdot \boldsymbol{\sigma})$. $\mathbf{S}$ is the unit vector of the magnetic moment, λ represents the strength of SOC on site i between the electron spin and its atomic orbital angular momentum $\mathbf{L}_i$ while $\boldsymbol{\sigma}$ is the vector of Pauli matrices. The impact of magnetism and SOC on the electronic structure can be tracked via the Dyson equation, which relates the new Green function g to the original one G:

$$g = G + Gvg = G + GvG + GvGvG + GvGvGvG + \dots\,, \tag{6}$$

where we performed Born iterations.

The expansion to second order $GvGvG$ gives rise to the two-site isotropic spin-mixing magnetoresistance (I-XMR), which does not require SOC as discussed in the main text. Using known

relations involving products of Pauli matrices: $(\mathbf{a}\cdot\boldsymbol{\sigma})(\mathbf{b}\cdot\boldsymbol{\sigma}) = (\mathbf{a}\cdot\mathbf{b})\,\sigma_0 + i(\mathbf{a}\times\mathbf{b})\cdot\boldsymbol{\sigma}$, we find that one of the terms contributing to the LDOS looks like

$$-\frac{1}{\pi}\mathrm{Im}\,\mathrm{Tr}\,G_{ij}(E)U_j(\mathbf{S}_j\cdot\boldsymbol{\sigma})G_{ji}(E)U_i(\mathbf{S}_i\cdot\boldsymbol{\sigma})G_{ii}(E), \tag{7}$$

yielding the general expansion

$$\mathrm{LDOS}_i^{(2)\ \text{I-XMR}}(E) \;=\; \sum_j f_{ij}^{(2)\ \text{I-XMR}}(E)(\mathbf{S}_i\cdot\mathbf{S}_j). \tag{8}$$

for the two-site two-spin scattering term with $f_{ij}^{(2)\ \text{I-XMR}}(E) = -\frac{2}{\pi}\mathrm{Im}\,\mathrm{Tr}\,G_{ij}(E)U_jG_{ji}(E)U_iG_{ii}(E)$. We also uncover isotropic SOC-independent higher-order scattering terms such as the 4-spin contribution:

$$\mathrm{LDOS}_i^{(4)\ \text{I-XMR}}(E) \propto \sum_{jkl} f_{ijkl}^{(4)\ \text{I-XMR}}(E)\left[(\mathbf{S}_i\cdot\mathbf{S}_j)(\mathbf{S}_k\cdot\mathbf{S}_l) - (\mathbf{S}_i\cdot\mathbf{S}_k)(\mathbf{S}_j\cdot\mathbf{S}_l) + (\mathbf{S}_i\cdot\mathbf{S}_l)(\mathbf{S}_j\cdot\mathbf{S}_k)\right], \tag{9}$$

which simplified to a biquadratic contribution if one limits the expansion to a two-site scattering event, as considered in the main text.

The third order term in the expansion of the Dyson equation gives rise to a chiral contribution when the scattering involves two magnetic sites and a third site carrying SOC. For instance, one of the terms looks like

$$\begin{aligned}
&-\frac{1}{\pi}\mathrm{Im}\,\mathrm{Tr}\,G_{ij}(E)v_jG_{jk}(E)v_k^{\mathrm{so}}G_{ki}(E)v_iG_{ii}(E)\\
=\;&-\frac{1}{\pi}\mathrm{Im}\,\mathrm{Tr}\,G_{ij}(E)U_j(\mathbf{S}_j\cdot\boldsymbol{\sigma})G_{jk}(E)\lambda_k(\mathbf{L}_k\cdot\boldsymbol{\sigma})G_{ki}(E)U_i(\mathbf{S}_i\cdot\boldsymbol{\sigma})G_{ii}(E)\\
=\;&-\frac{2}{\pi}\mathrm{Re}\,\mathrm{Tr}\,G_{ki}(E)U_iG_{ii}(E)G_{ij}(E)U_jG_{jk}(E)\lambda_k\,\mathbf{L}_k\cdot(\mathbf{S}_i\times\mathbf{S}_j)\,.
\end{aligned} \tag{10}$$

Thus in general, we expect a chiral dependence of the LDOS:

$$\text{LDOS}_i^{\text{C-XMR}}(E) \;=\; \sum_j \mathbf{f}_{ij}^{\text{C-XMR}}(E)\cdot(\mathbf{S}_i\times\mathbf{S}_j), \tag{11}$$

which is linear with SOC similarly to the Dzyaloshinskii-Moriya interaction (DMI) [2,3]. $\mathbf{f}_{ij}^{\text{C-XMR}}(E)$ is the analog of the DMI vector and thus its direction obeys the same symmetry rules and its existence requires broken inversion symmetry.

Interestingly, a fourth-order scattering term (second-order scattering at magnetic and SOC sites), quadratic with respect to SOC, leads to the usual anisotropic magnetoresistance contribution, if the scattering occurs on the same site (local contribution). A non-local contribution is however possible, which gives rise to the anisotropic XMR (X-AMR). The conventional AMR can be expressed as:

$$\text{LDOS}_i^{\text{AMR}}(E) \;=\; \left(\mathbf{S}_i\cdot\mathbf{f}_i^{\text{X-AMR}}(E)\right)^2 , \tag{12}$$

where $\mathbf{f}^{\text{X-AMR}}$ represents the anisotropy field. This leads to the usual phenomenological form $\cos^2\theta_i$ characterizing the AMR signal, where theta is the polar angle of the magnetic moment at site i with respect to $\mathbf{f}$.

Besides the local contribution, there are various possible non-local terms shaping the total AMR. Their form emerging from a two-site scattering event can be written as:

$$\text{LDOS}_i^{\text{X-AMR}}(E) \;=\; \sum_{j\neq i}\left(\mathbf{S}_i\cdot\mathbf{f}_j^{\text{X-AMR}}(E)\right)\left(\mathbf{S}_j\cdot\mathbf{f}_i^{\text{X-AMR}}(E)\right). \tag{13}$$

As it can be recognized, the angular dependence of the X-AMR is different from the AMR one: $\cos\theta_i\cos\theta_j$ instead of $\cos^2\theta_i$.

Finally, we get a correction to the I-XMR contribution, which is quadratic with SOC, dubbed here anisotropic XMR (Ani-XMR):

$$\text{LDOS}_i^{\text{Ani-XMR}}(E) \propto \sum_{j\neq i} f_{ij}^{\text{Ani-XMR}}(E)\,(\mathbf{S}_i \cdot \mathbf{S}_j)\,, \tag{14}$$

where f^{AniXMR} is a scalar quantity that depends on SOC.

Supplementary Note 3 - XMR efficiency without SOC – I-XMR.

The XMR efficiency is obtained by taking the difference of the LDOS decaying into vacuum (probed by the STM tip) between the two cases: non-collinear (NC) versus collinear (C) spin states. The difference is then normalized by the collinear LDOS:

$$\text{XMR}^{\gamma}(E) = \frac{\text{LDOS}_{\text{NC}}^{\gamma}(E) - \text{LDOS}_{\text{C}}(E)}{\text{LDOS}_{\text{C}}(E)} \tag{15}$$

where γ indicates the helicity of the skyrmion.

Following Supplementary Equation 8 and 9, the XMR effect without SOC contribution at a position i is found to depend on the dot-product with the surrounding 6 nearest neighbor spins,

$$\text{I-XMR}(E) \approx \gamma_{\text{I-XMR}}^{\text{2-spin}}(E) \sum_{j\in\text{NN}} \mathbf{S}_i \cdot \mathbf{S}_j + \gamma_{\text{I-XMR}}^{\text{4-spin}}(E) \sum_{j\in\text{NN}} (\mathbf{S}_i \cdot \mathbf{S}_j)^2 - 6\left(\gamma_{\text{I-XMR}}^{\text{2-spin}}(E) + \gamma_{\text{I-XMR}}^{\text{4-spin}}(E)\right)\,, \tag{16}$$

where $\gamma_{\text{I-XMR}}$ describes the I-XMR weight that one can extract from a fit of the measured transport spectra.

We collect the sum of the dot products of each of the magnetic moments with their respec-

tive 6 nearest neighbors and plot the corresponding atomically resolved I-XMR in Supplementary Figure 1 at different bias voltages. The red circles represent the values obtained from our *ab initio* calculations for each atom within the Néel-type skyrmion with diameter $D_{Sk} \approx 2.2\,\mathrm{nm}$. The straight lines represent a good fit to the *ab initio* data, which provide a reasonable estimate of $\gamma_{\text{I-XMR}}$.

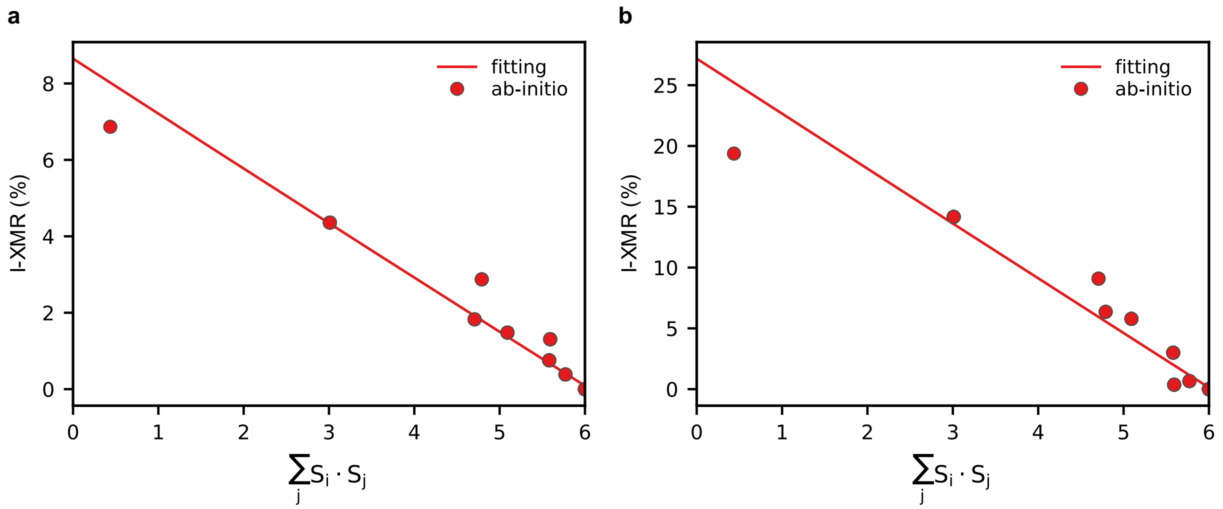

Supplementary Figure 1: Fit of the I-XMR weight. First-principles results (circles) of the I-XMR effect at a particular tunneling energy **a** $eV_{\text{bias}} = 0.21\,\text{eV}$ and **b** $eV_{\text{bias}} = 0.52\,\text{eV}$ as function of the sum of the dot product of nearest neighboring spin moments. These results are then fitted following Supplementary Equation 16 (solid line).

Supplementary Note 4 - Chiral spin-mixing magnetoresistance – C-XMR.

Following Supplementary Equation 11, the C-XMR efficiency can be written as

$$\text{C-XMR}_i(E) \approx \gamma_{\text{C-XMR}}(E) \sum_{j \in \text{NN}} \hat{\mathbf{c}}_{ij} \cdot \mathbf{S}_i \times \mathbf{S}_j \,, \tag{17}$$

assuming the contribution of nearest neighbors to site i and where $\hat{\mathbf{c}}$ is a chirality vector parallel to $\mathbf{f}^{\text{C-XMR}}$. $\hat{\mathbf{c}}$ obeys the same symmetry rules dictating the direction of the DMI. In the particular case studied in the paper, the in-plane components of $\hat{\mathbf{c}}$ follow those of the DM vector. The z-component is however unknown. Thus, we choose here to modulate that component by a factor of α with respect to that of the DM vector (see Supplementary Figure 2a-c).

In order to quantify $\gamma_{\text{C-XMR}}$, we proceed to a fit similarly to what was done in the previous Note. Here, however, the ab-initio obtained data will be shifted laterally depending on α as plotted in Supplementary Figure 2d-e. Since the z-component of the DMI vector is rather small, there are no significant differences among the data points associated with the three values considered for α: 1.0 (red dots), 0.5 (blue dots) and 0.0, i.e. with purely in-plane $\hat{\mathbf{c}}$ (green circles). Once more the linear fit seems reasonable. If there is mismatch, it can be assigned to the chiral scattering contribution from second nearest neighbors of higher-order chiral contributions.

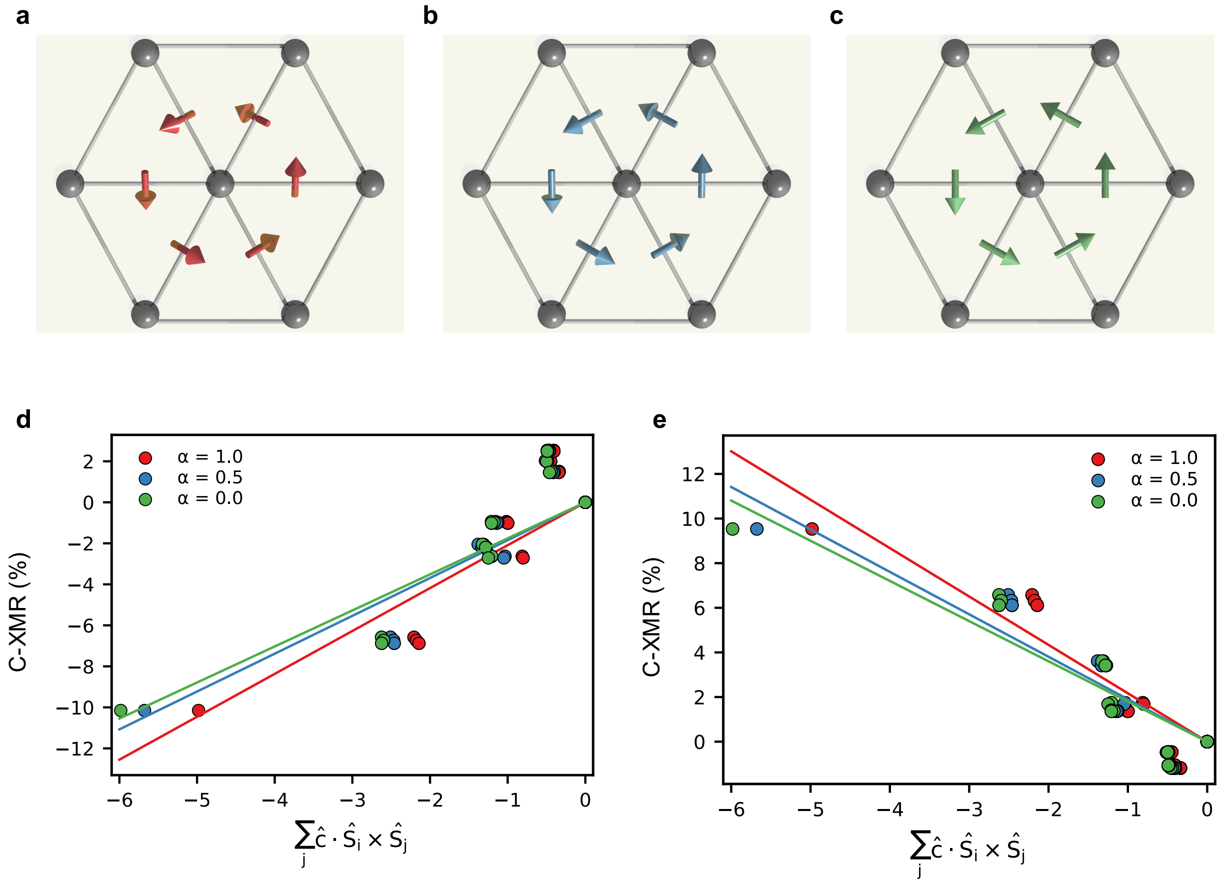

Supplementary Figure 2: Fit of the C-XMR weight. he $\hat{c}$ vector follows the orientation of the DMI-vector with the z-component scaled by the factor **a** $\alpha = 1.0$ (red), **b** $\alpha = 0.5$ (blue) and **c** $\alpha = 0.0$ (green). First-principles results (circles) of the C-XMR effect at a particular tunneling energy **d** $eV_{\text{bias}} = 0.61\,\text{eV}$ and **e** $eV_{\text{bias}} = 0.89\,\text{eV}$ as function of the cross product of $\mathbf{S}_i$ with the surrounding 6 nearest neighbor spins. These results are then fitted (solid lines) according to Supplementary Equation 17, considering the factor $\alpha = 1.0$ (red), $\alpha = 0.5$ (blue) and $\alpha = 0.0$ (green).

Supplementary Note 5 - Anisotropic magnetoresistances – AMR, X-AMR and Ani-XMR.

Starting with Supplementary Equation 13-14 and limiting the scattering contributions to the nearest neighbors, the total AMR, which is biquadratic with SOC, at a site i contains three contributions:

$$\begin{aligned} \text{AMR}^i_{\text{total}}(E) &\approx \gamma_{\text{AMR}}(E)\,\cos^2\theta_i + \gamma_{\text{X-AMR}}(E)\sum_{j\in\text{NN}}\cos\theta_i\cos\theta_j + \gamma_{\text{Ani-XMR}}(E)\sum_{j\in\text{NN}}\mathbf{S}_i\cdot\mathbf{S}_j \\ &- \left(\gamma_{\text{AMR}}(E) + 6\gamma_{\text{X-AMR}}(E) + 6\gamma_{\text{Ani-XMR}}(E)\right)\,, \end{aligned} \tag{18}$$

where θ_i is the polar angle associated with the magnetic moment at site i.

In general the non-local contribution, X-AMR, provides the largest weight to the total AMR. An example of a fitting procedure to extract the various weights is illustrated in Supplementary Figure 3. We proceed to the different fits in a sequential fashion. We start by extracting $\gamma_{\text{X-AMR}}$ (Supplementary Figure 3a,d). The rest of the signal $\text{AMR}_{\text{total}} - \text{X-AMR}$ is then fitted to obtain γ_{AMR} (Supplementary Figure 3b,e). Finally, we conclude with the last weight $\gamma_{\text{Ani-XMR}}$ (Supplementary Figure 3c,f).

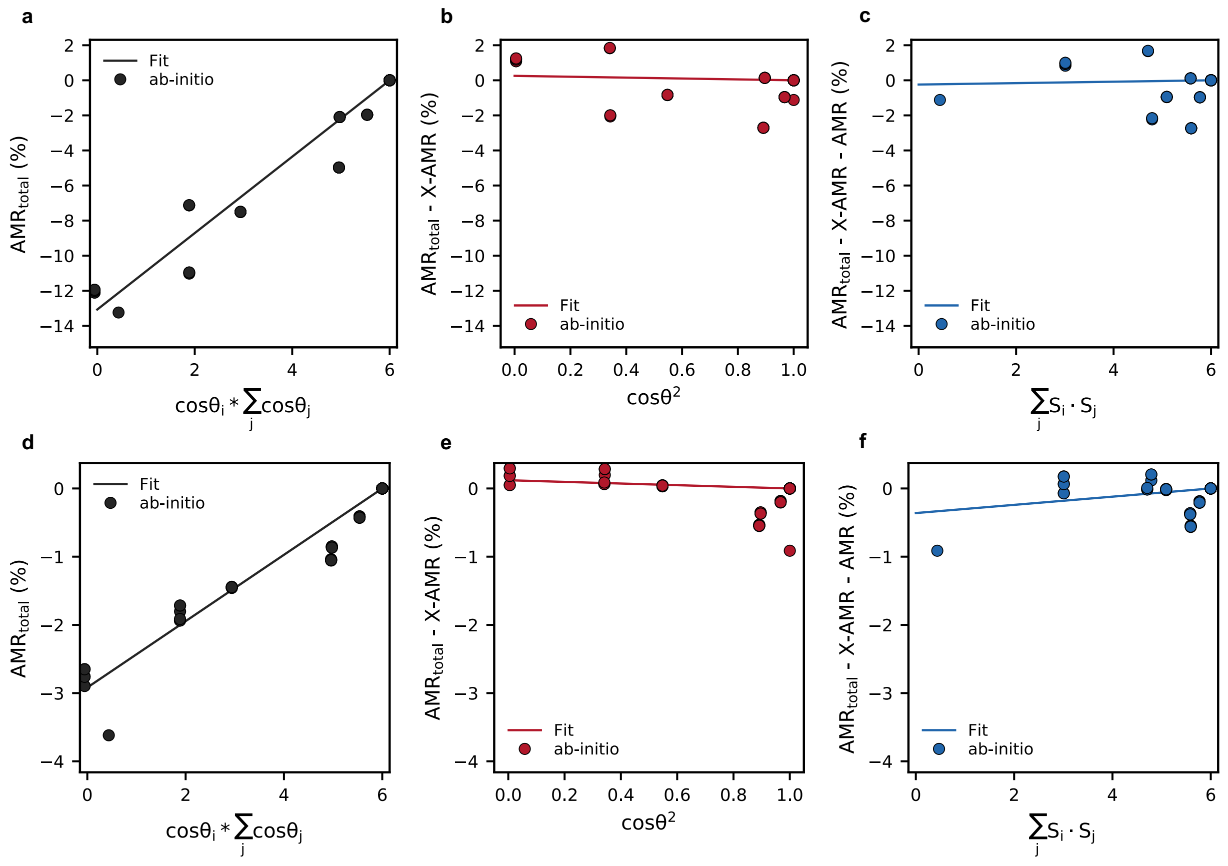

Supplementary Figure 3: Fit of the family of AMR weights. First-principles results (black circles) of the total AMR at site i at a tunneling energy **a** $eV_{\text{bias}} = -1.29\,\text{eV}$ (**d** $eV_{\text{bias}} = 1.01\,\text{eV}$) as function of the sum of products $\cos\theta_i \cos\theta_j$ with the surrounding 6 nearest neighbor spins. These results are then fitted to extract $\gamma_{\text{X-AMR}}$ (solid black line). The remaining local (conventional) AMR and anisotropic XMR weights are obtained in **b** (**e**) and **c** (**f**) respectively.

Supplementary Note 6 - Magnetoresistances associated with various skyrmionic structures.

The various MR patterns hosted by Neel with helicity $\gamma = \pi$, Bloch skyrmions and antiskyrmions at 0.61 eV are illustrated in Supplementary Figures 4-6, respectively. One can clearly see that the C-XMR effect can be used to distinguish skyrmionic structures while all other MRs are identical since they are independent of the chirality/helicity of the spin-textures.

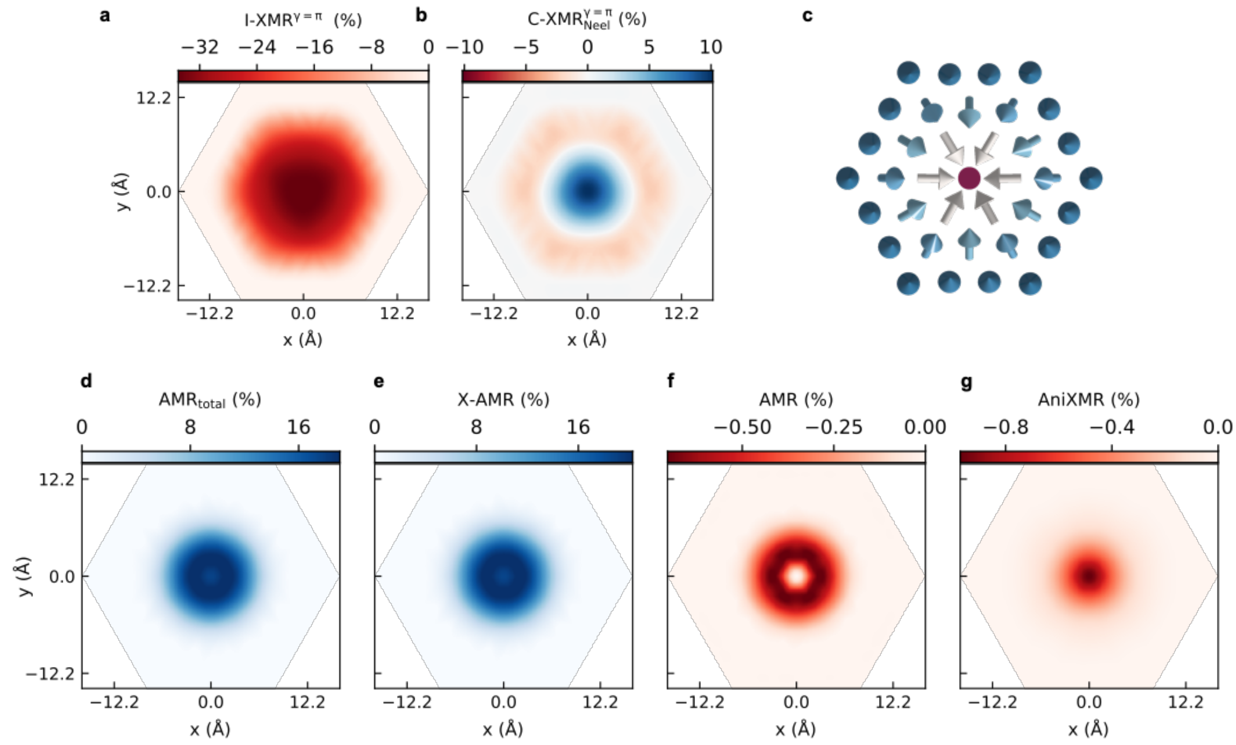

Supplementary Figure 4: Magnetoresistance patterns characterizing a Néel skyrmion with $\gamma = \pi$. a-b Spin-orbit independent XMR pattern compared to the one emerging from C-XMR as obtained at the energy bias of $eV_{\text{bias}} = 0.61\,\text{eV}$. **c** Néeel-type skyrmion with helicity $\gamma = \pi$. **d** Total AMR signal decomposed into the **e** X-AMR, **f** conventional AMR, and **g** ani-XMR components.

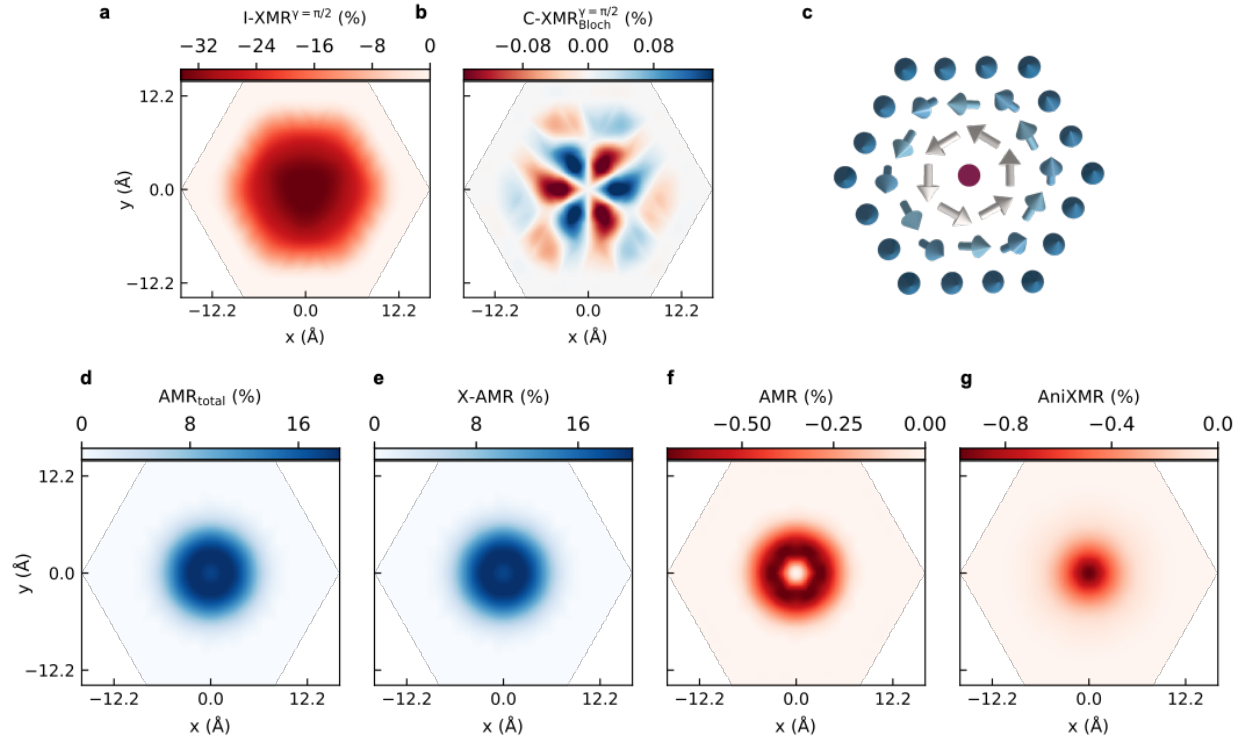

Supplementary Figure 5: Magnetoresistance patterns characterizing a Bloch skyrmion. a-b Spin-orbit independent XMR pattern compared to the one emerging from C-XMR as obtained at the energy bias of $eV_{\text{bias}} = 0.61\,\text{eV}$. **c** Bloch-type skyrmion with helicity $\gamma = \pi/2$. **d** Total AMR signal decomposed into the **e** X-AMR, **f** conventional AMR, and **g** ani-XMR components.

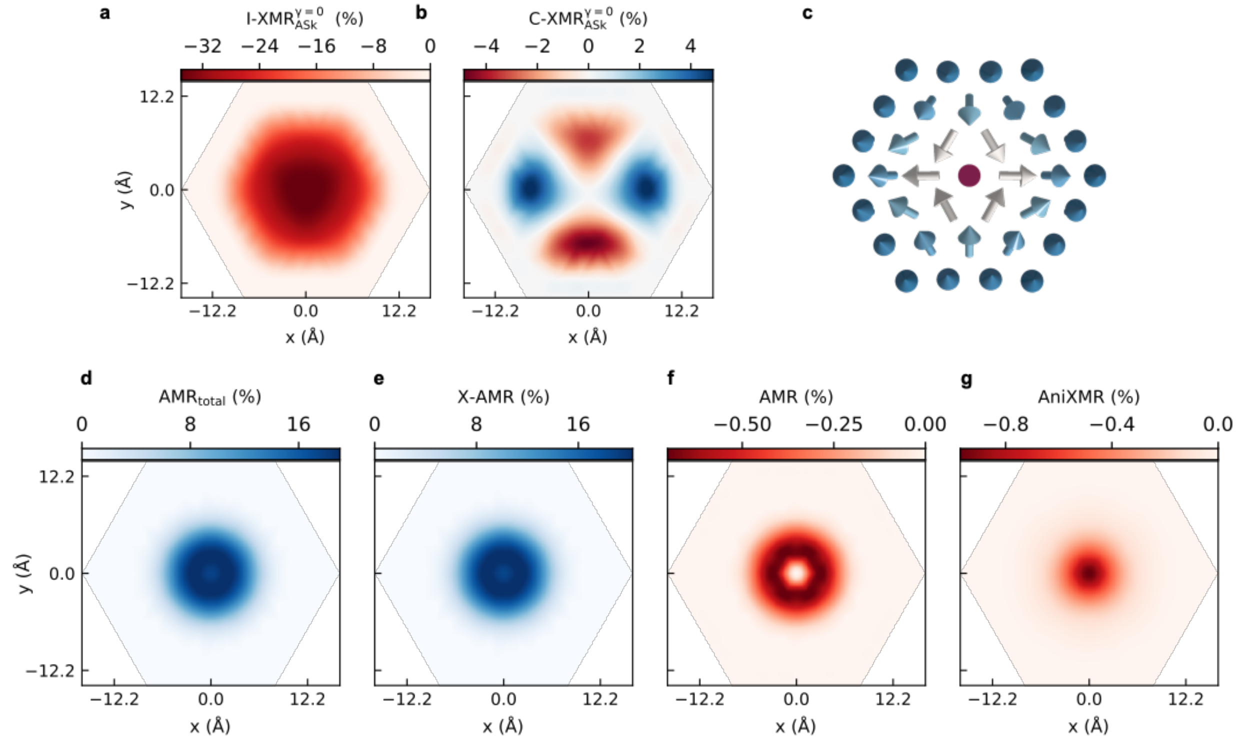

Supplementary Figure 6: Magnetoresistance patterns characterizing an Anti-skyrmion. a-b Spin-orbit independent XMR pattern compared to the one emerging from C-XMR as obtained at the energy bias of $eV_{\text{bias}} = 0.61\,\text{eV}$. **c** Anti-skyrmion with helicity $\gamma = 0$. **d** Total AMR signal decomposed into the **e** X-AMR, **f** conventional AMR, and **g** ani-XMR components.

Supplementary References

1. Nagaosa, N. & Tokura, Y. Topological properties and dynamics of magnetic skyrmions. *Nat. Nanotech.* **8**, 899–911 (2013).

2. Dzyaloshinsky, I. A thermodynamic theory of “weak” ferromagnetism of antiferromagnetics. *J. Phys. Chem. Sol.* **4**, 241 – 255 (1958).

3. Moriya, T. Anisotropic superexchange interaction and weak ferromagnetism. *Phys. Rev.* **120**, 91–98 (1960).
